# *Mycobacterium smegmatis* PhoU Proteins Have Overlapping Functions in Phosphate Signaling and Are Essential

**DOI:** 10.3389/fmicb.2017.02523

**Published:** 2017-12-18

**Authors:** Alyssa M. Brokaw, Benjamin J. Eide, Michael Muradian, Joshua M. Boster, Anna D. Tischler

**Affiliations:** Department of Microbiology and Immunology, University of Minnesota, Minneapolis, MN, United States

**Keywords:** Pst system, phosphate, PhoU, RegX3, tuberculosis, persister, antibiotic tolerance, polyphosphate

## Abstract

Many bacteria regulate gene expression in response to phosphate availability using a two-component signal transduction system, the activity of which is controlled by interaction with the Pst phosphate specific transporter and a cytoplasmic protein PhoU. *Mycobacterium tuberculosis*, the causative agent of tuberculosis, requires its phosphate sensing signal transduction system for virulence and antibiotic tolerance, but the molecular mechanisms of phosphate sensing remain poorly characterized. *M. smegmatis* serves as a model for studying mycobacterial pathogens including *M. tuberculosis. M. smegmatis* encodes two proteins with similarity to PhoU, but it was unknown if both proteins participated in signal transduction with the phosphate-responsive SenX3-RegX3 two-component system. We constructed *phoU* single and double deletion mutants and tested expression of genes in the RegX3 regulon. Only the Δ*phoU1*Δ*phoU2* mutant exhibited constitutive activation of all the RegX3-regulated genes examined, suggesting that *M. smegmatis* PhoU1 and PhoU2 have overlapping functions in inhibiting activity of the SenX3-RegX3 two-component system when phosphate is readily available. The Δ*phoU1*Δ*phoU2* mutant also exhibited decreased tolerance to several anti-tubercular drugs. However, a complex plasmid swapping strategy was required to generate the Δ*phoU1*Δ*phoU2* mutant, suggesting that either *phoU1* or *phoU2* is essential for *in vitro* growth of *M. smegmatis*. Using whole-genome sequencing, we demonstrated that all five of the Δ*phoU1*Δ*phoU2* mutants we isolated had independent suppressor mutations predicted to disrupt the function of the Pst phosphate transporter, suggesting that in the absence of the PhoU proteins phosphate uptake by the Pst system is toxic. Collectively, our data demonstrate that the two *M. smegmatis* PhoU orthologs have overlapping functions in both controlling SenX3-RegX3 activity in response to phosphate availability and regulating phosphate transport by the Pst system. Our results suggest that *M. smegmatis* can serve as a tractable model for further characterization of the molecular mechanism of phosphate sensing in mycobacteria and to screen for compounds that would interfere with signal transduction and thereby increase the efficacy of existing anti-tubercular antibiotics.

## Introduction

In many bacteria, the transcriptional response to phosphate limitation is mediated by a two-component signal transduction system whose activity is controlled by a poorly understood interaction with the Pst (phosphate specific transport) inorganic phosphate (P_i_) uptake system (Lamarche et al., 2008; Hsieh and Wanner, 2010). In the model organism *Escherichia coli*, the two-component system PhoR-PhoB is activated only when the bacteria are P_i_ starved; in P_i_-replete conditions, the Pst P_i_ uptake system inhibits activation of PhoR-PhoB (Wanner, 1996). Although the precise molecular mechanism of inhibition is not known, null mutations in any component of the Pst system result in constitutive activation of PhoR-PhoB and constitutive expression of the P_i_-starvation responsive Pho regulon (Wanner, 1996). Like *E. coli*, mycobacteria, including the human pathogen *Mycobacterium tuberculosis*, encode a two-component system SenX3-RegX3 that is activated by P_i_ limitation and that requires a functional Pst P_i_ uptake system for P_i_ sensing (Glover et al., 2007; Rifat et al., 2009; Tischler et al., 2013). In *M. tuberculosis* and other pathogens, both the Pst system and the two-component system are essential for virulence (Parish et al., 2003; Lamarche et al., 2008; Tischler et al., 2013). It is therefore of interest to understand at the molecular level how the Pst P_i_ uptake and two-component signal transduction systems interact, since small molecules targeting this interaction could have robust and broad spectrum antimicrobial activity.

In the *E. coli* model, an additional protein named PhoU is involved in P_i_ sensing. PhoU is not required for P_i_ uptake by the Pst system (Steed and Wanner, 1993), though it may regulate Pst system P_i_ transport activity (Rice et al., 2009). Mutation of *phoU*, like mutation of any component of the Pst system, results in constitutive activation of the PhoR-PhoB two-component system and expression of the Pho regulon in P_i_-replete conditions (Li and Zhang, 2007). Recent evidence suggests that PhoU communicates signals from the Pst system to PhoR-PhoB via direct protein-protein interactions between PhoU and PstB, the Pst system ATPase, and between PhoU and the PhoR sensor kinase (Gardner et al., 2014). *phoU* mutants in *E. coli* and other bacterial species accumulate polyphosphate (PolyP), a linear polymer of P_i_ linked by high energy phosphoanhydride bonds, suggesting a possible role of PhoU in regulation of cellular P_i_ metabolism (Morohoshi et al., 2002; de Almeida et al., 2015; Lubin et al., 2016). Finally, *E. coli phoU* is necessary for the development of persisters, a sub-population that is phenotypically tolerant to antibiotics (Li and Zhang, 2007; Maissonneuve and Gerdes, 2014). However, the molecular mechanism by which PhoU promotes persister formation is unknown.

Mycobacteria are unusual in that they encode multiple copies of genes with similarity to *E. coli phoU*. In *M. tuberculosis*, the *phoU* orthologs were named *phoY1* (*rv3301c*) and *phoY2* (*rv0821c*). *M. tuberculosis* may have evolved two PhoU orthologs since it also encodes two complete Pst P_i_ uptake systems (Braibant et al., 2000), though only one of these systems functions in P_i_ sensing and signal transduction with SenX3-RegX3 (Tischler et al., 2013, 2016). In contrast, the fast-growing saprophytic species *Mycobacterium smegmatis* has a single Pst system, but encodes two *phoU* orthologs. One ortholog, which we have named *phoU1* (*Msmeg_5776*), is encoded adjacent to the *M. smegmatis pstSCAB* operon. The second ortholog, which we have named *phoU2* (*Msmeg_1605*), is encoded in a region of the genome distant from this locus. The PhoU1 and PhoU2 proteins exhibit 82% sequence similarity and are both encoded separately from the SenX3-RegX3 two-component system (*Msmeg_0936* and *Msmeg_0937*). Previous reports have suggested that PhoY2 of *M. tuberculosis* is important for development of antibiotic tolerant persister variants (Shi and Zhang, 2010) and that PhoY2 in the related pathogen *Mycobacterium marinum* influences P_i_ homeostasis, energy and redox balance (Wang et al., 2013). However, we recently demonstrated that *M. tuberculosis* PhoY1 and PhoY2 function redundantly to control activity of the SenX3-RegX3 system and promote persister formation (Namugenyi et al., 2017). It is not known which of the *M. smegmatis* PhoU orthologs participates in P_i_ sensing and signal transduction or whether they contribute to antibiotic tolerance.

We hypothesized that the *M. smegmatis* PhoU orthologs would exhibit redundant function in transcriptional regulation and antibiotic tolerance, similar to the *M. tuberculosis* PhoY proteins. We generated deletions of *phoU1* and *phoU2* in *M. smegmatis* and evaluated expression of RegX3-regulated genes and antibiotic sensitivity. We found that it was necessary to delete both *phoU1* and *phoU2* to observe complete activation of the RegX3 regulon in P_i_-rich conditions, suggesting partial functional redundancy between *M. smegmatis* PhoU1 and PhoU2. However, the Δ*phoU1*Δ*phoU2* mutant was difficult to construct suggesting that PhoU1 or PhoU2 are jointly essential for *M. smegmatis* viability. Using whole-genome sequencing, we demonstrated that all five of the Δ*phoU1ΔphoU2* mutants that we isolated have independent mutations predicted to disrupt the function of the Pst transporter. Collectively, our data suggest that the *M. smegmatis* PhoU proteins have overlapping functions in both controlling activation of SenX3-RegX3 and regulating P_i_ transport by the Pst system.

## Materials and Methods

### Bacterial Culture Conditions

*M. smegmatis* mc^2^155 and derivative strains were grown at 37°C in Middlebrook 7H9 (Difco) liquid culture medium supplemented with 10% albumin-dextrose-saline (ADS), 0.5% glycerol and 0.1% Tween-80 or on Luria Broth (LB) agar solid culture medium. Frozen stocks were prepared by growing cultures to mid-exponential phase (OD_600_ of 0.6–0.8), adding glycerol to 15% final concentration, and storing aliquots at -80°C. Antibiotics were used at the following concentrations: kanamycin (Kan) 15 μg/ml, hygromycin (Hyg) 50 μg/ml, rifampicin (RIF) 200 μg/ml, isoniazid (INH) 50 μg/ml, ethionamide (ETH) 200 μg/ml, ethambutol (ETB) 5 μg/ml.

### Cloning

All plasmids used for cloning and strain construction are listed in Supplementary Table S1. Constructs for deletion of *phoU1* (*Msmeg_5776*) or *phoU2* (*Msmeg_1605*) in *M. smegmatis* were generated in the allelic exchange vector pJG1100 (Kirksey et al., 2011). Genomic regions 500–800 bp upstream and downstream of the genes to be deleted were PCR-amplified from *M. smegmatis* mc^2^155 genomic DNA using the oligonucleotides listed in Supplementary Table S2. Reverse primers for amplification of the upstream regions were designed with an AvrII restriction site in-frame with the translation start codon; corresponding forward primers for amplification of the downstream regions were designed with an AvrII restriction site in-frame with the stop codon. PCR products were cloned in pCR2.1-TOPO (Invitrogen) and sequenced. Upstream and downstream regions were removed from pCR2.1 by restriction with PacI/AvrII and AvrII/AscI, respectively and ligated together in pJG1100 between the PacI and AscI sites to generate the in-frame deletion constructs pBE101 (Δ*phoU1*), pBE102 (Δ*phoU2*).

Vectors for complementation of the *phoU* deletions were constructed in the episomal plasmid pMV261 under the control of the vector-encoded strong constitutive *hsp60* promoter. The *M. smegmatis phoU1* and *phoU2* genes were PCR-amplified using the primers indicated in Supplementary Table S2, cloned in pCR2.1-TOPO and sequenced. The cloned genes were removed from pCR2.1 by restriction with EcoRI and HindIII and ligated in similarly digested pMV261 to generate pMV*phoU1* and pMV*phoU2*.

For construction of Tet-inducible *phoU1*, the *phoU1* gene was PCR-amplified using the primers indicated in Supplementary Table S2, cloned in pCR2.1-TOPO and sequenced. *phoU1* was removed from pCR2.1 by restriction with HindIII and EcoRI and ligated in similarly digested pTIC10a or pJT6a to generate pTIC*phoU1* and pJT*phoU1*.

### Strain Construction

*M. smegmatis* Δ*phoU1* and Δ*phoU2* deletion mutants were generated by a two-step homologous recombination method of allelic exchange, as previously described (Upton and McKinney, 2007) except that LB agar medium containing Kan and Hyg and LB agar medium containing 5% sucrose were used for selection of transformants and counter-selection of the pJG1100 vector, respectively. Integration of the vectors was confirmed with the following primer pairs, listed in Supplementary Table S2: Δ*phoU1* upstream 5776F3/5776R4; Δ*phoU1* downstream 5776F4/5776R3; Δ*phoU2* upstream 1605F3/1605R4; Δ*phoU2* downstream 1605F4/1605R3. Identification of deletion mutants was done with the following primer pairs: Δ*phoU1* 5776F3/5776R3; Δ*phoU2* 1605F3/1605R3. Complemented strains were constructed by electroporating the corresponding deletion mutant with the complementation plasmid pMV*phoU1* or pMV*phoU2*, and selecting on LB containing Kan.

For construction of the Δ*phoU1ΔphoU2* double deletion mutant, the Δ*phoU2* mutant was electroporated with pTIC*phoU1* and transformants were selected on LB containing Kan. The resulting strain was then electroporated with the pBE101 (Δ*phoU1*) allelic exchange vector and transformants were selected on LB containing Kan and Hyg. Integration of the Δ*phoU1* vector in the transformants was confirmed by PCR as described above. The resulting strain was then grown overnight in 7H9 medium containing 50 ng/ml anhydrotetracycline (ATc) to induce expression from the pTIC*phoU1* construct and subsequently plated on LB containing 5% sucrose and 100 ng/ml ATc to counterselect the pBE101 allelic exchange vector. Individual Hyg^S^ colonies were tested for the Δ*phoU1* deletion by PCR using the 5776F3/5776R3 primer pair. The resulting Δ*phoU1ΔphoU2*pTIC*phoU1* strain was then electroporated with pJT6a and transformants were selected on LB containing Hyg. Individual Hyg^R^ transformants were patched to LB Kan and LB Hyg. Those that were Hyg^R^ and Kan^S^ were selected for PCR analysis with primers pTfor and pTIC6a_R that amplify a 1.3 kbp fragment from pTIC*phoU1* and a 500 bp fragment from pJT6a. Isolates from which only the 500 bp product was amplified were tested for the deletions of *phoU1* and *phoU2* by PCR as described above.

### Growth Curves

Cultures of *M. smegmatis* grown in complete 7H9 medium to mid-logarithmic phase were diluted to OD_600_ 0.01 in 7H9 and 200 μl were inoculated in a 96 well plate (polystyrene rounded square well, Fisherbrand). Cultures were incubated at 37°C with shaking and OD_600_ was monitored hourly for 36 h using a Synergy H1 hybrid reader (BioTek).

### Analysis of Clumping Phenotype

Cultures of *M. smegmatis* were grown at 37°C with shaking in either 14 ml polystyrene snap-cap tubes (Falcon) in 5 ml complete 7H9 medium to assess pellicle formation or a polystyrene flat-bottom 12-well plate (Falcon) in 4 ml complete 7H9 medium to test clumping. After 72 h of incubation, the tubes and plate were imaged.

### Determination of Colony Sizes

Isolated single colonies of *M. smegmatis* were grown on LB agar for 4 days at 37°C. Plates were imaged with a FluorChem FC3 (Cell Biosciences) using the white light setting. Colony area for 50 representative colonies was measured on each of three plates grown from independent biological replicates using ImageJ.

### Quantitative RT-PCR (qRT-PCR)

Bacteria were grown to mid-exponential phase (OD_600_ 0.5) in 7H9 broth and RNA was extracted as described (Tischler et al., 2013). Equivalent amounts of total RNA were treated with Turbo DNase (Ambion) and reverse transcribed to cDNA with the Transcriptor First Strand cDNA Synthesis Kit (Roche) using random hexamers for priming and the following cycling conditions: 10 min at 25°C for annealing, 60 min at 50°C for extension, 5 min at 85°C for heat inactivation. cDNA was stored at -20°C until real-time PCR reactions were performed. Quantitative Real Time PCR reactions were prepared with 2× FastStart Sybr Green Master Mix (Roche), 2 μl cDNA, and 0.3 μM primers and were run in absolute quantification mode on a LightCycler 480 (Roche). PCR cycling conditions were: 95°C 10 min; 45 cycles of 95°C for 10 s, 60°C for 20 s, 72°C for 20 s with data collection once per cycle during the extension phase; one cycle of 95°C for 5 s, 60°C for 1 min, 97°C for 15 s with a 0.11°C/s ramp rate during the final denaturation to generate melting curves for confirmation of product specificity. Mock reactions (no RT) were performed on each sample to confirm the absence of genomic DNA contamination. Cp values were converted to copy numbers using standard curves for each gene. Target cDNA was internally normalized to *sigA* cDNA. Primers for real-time quantitative reverse transcriptase (RT) PCR used in this study are listed in Supplementary Table S3. Primers were designed using the Roche online Universal ProbeLibrary assay design center tool and were tested in standard PCR reactions using 100 *M. smegmatis* genome equivalents as template.

### Persister Assay

Cultures of *M. smegmatis* grown in complete 7H9 medium overnight to late-logarithmic phase were diluted to OD_600_ 0.05 in 20 ml complete 7H9 and antibiotics were added. Cultures were incubated at 37°C with aeration and viable CFU were determined at 0, 8, 24, 48, and 72 h by plating serially diluted cultures on LB agar medium. CFU were enumerated after 3–4 days of incubation at 37°C.

### Minimal Inhibitory Concentration (MIC) Assay

Cultures of *M. smegmatis* grown in complete 7H9 medium to mid-logarithmic phase (OD_600_ of 0.5) were diluted to OD_600_ of 0.002 in fresh complete 7H9 medium. Antibiotics were added to 2 ml aliquots of the dilute culture in twofold decreasing concentrations with the highest concentration at 10x the MIC for wild-type *M. smegmatis* mc^2^155 (RIF 200 μg/ml, INH 50 μg/ml, EMB 5 μg/ml, ETH 200 μg/ml). Cultures were incubated for 48 h at 37°C with shaking and the OD_600_ was determined. The MIC_90_ was the concentration of antibiotic that inhibited 90% of the growth of a no drug control.

### Alkaline Phosphatase Assay

Cultures of *M. smegmatis* were grown in complete 7H9 medium to mid-logarithmic phase (OD_600_ 0.4–0.8) and centrifuged at low speed (150 × *g* for 5 min) to remove large clumps. The OD_600_ of the de-clumped culture was determined, and 0.5 ml of bacteria from this culture were pelleted by centrifugation (16000 × *g* for 10 min). The culture supernatant was removed and bacteria were resuspended in 0.1 ml 1M Tris buffer, pH 8.1 ml of 2 mM *p*-nitrophenyl phosphate was added and samples were incubated at 37°C in the dark for 10 min. Bacteria were removed by centrifugation and 1 ml of the supernatant was transferred to a cuvette. The OD_420_ of the sample was measured using 2 mM *p*-nitrophenyl phosphate as a blank. Alkaline phosphate units were calculated as (1000 × OD_420_)/(minutes of incubation × OD_600_ × 0.5 ml).

### Polyphosphate Extraction and Quantification

Cultures of *M. smegmatis* were grown to mid-logarithmic phase (OD_600_ 0.5–1.0) in complete 7H9. Cells from 20 ml of culture were pelleted (4700 × *g* for 15 min), then resuspended and lysed in 4 M guanidine isothiocyanate, 50 mM Tris-HCl (pH 7) at 95°C for 30 min. Total protein was quantified using 10 μl of the lysed sample (Pierce BCA Protein Concentration Assay, Thermo Scientific). PolyP was isolated from the remaining cell lysate using glassmilk (GeneClean) and quantified by binding to toluidine blue O (Sigma) dye solution (6 mg/L in 40 mM acetic acid) as previously described (Namugenyi et al., 2017).

### Genomic DNA Extraction

*M. smegmatis* strains were grown to mid-logarithmic phase (OD_600_ 0.5–0.8) and bacteria from 20 ml of culture were pelleted by centrifugation (2850 × *g* for 10 min). Genomic DNA was extracted by the cetyltrimethylammonium bromide (CTAB) – lysozyme method as described (Larsen et al., 2007) except that bacteria were lysed by incubation with lysozyme for 3 h at 37°C. DNA was resuspended in 100 μl TE buffer and stored at 4°C overnight. Concentration and purity of the genomic DNA were determined using a Nanodrop spectrophotometer (Thermo Scientific).

### Whole-Genome Sequencing

Genomic DNA extracted from *M. smegmatis* mc^2^155 and five Δ*phoU1ΔphoU2* isolates was diluted to 12 ng/μl in 25 μl DEPC-treated water and submitted to the University of Minnesota Genomics Center. Whole-genome sequencing libraries were created using the TruSeq Nano Library Preparation Kit (Illumina). Libraries were processed using the 350 bp shearing protocol according to the manufacturer’s instructions. Libraries were multiplexed and sequenced on 0.25 lane of a HiSeq 2500 (Illumina) in high-output mode using the cBot HiSeq PE Cluster Kit v4 (Illumina) to generate clusters, and HiSeq SBS Kit, v4 chemistry (Illumina) with paired-end 125 bp reads. Image analysis and base calling were done using the Illumina software package RTA v 1.18.62. De-multiplexing and fastq file creation were performed using bcl2fastq v2.17.1.14. Total sequence yields (in Gb) were: mc^2^155 = 13.6; Δ*phoU1ΔphoU2* #504 = 5.1; Δ*phoU1ΔphoU2* #518 = 9.1; Δ*phoU1ΔphoU2* #521 = 4.4; Δ*phoU1ΔphoU2* #625 = 8.4; Δ*phoU1ΔphoU2* #664 = 4.8. Sequences from mc^2^155 were aligned to the reference *M. smegmatis* mc^2^155 sequence in Geneious version 10.0.9 software^1^ (Kearse et al., 2012) using the Geneious mapper to generate a Tischler lab WT mc^2^155 consensus sequence. Sequences from each of the five Δ*phoU1ΔphoU2* mutants were then aligned to the WT mc^2^155 consensus using the Geneious mapper to identify variants. Single nucleotide polymorphisms (SNPs) and short duplications in *Msmeg_1387, pstS*, *pstC*, and *pstB* were confirmed by PCR amplification and sequencing using the primers listed in Supplementary Table S2.

### Statistical Analysis

Student’s unpaired *t*-test (two-tailed) was used for pairwise comparisons between WT and mutant strains of *M. tuberculosis. P* values were calculated using GraphPad Prism 5.0 software (GraphPad Software, Inc.). *P* values < 0.05 were considered significant.

## Results

### *M. smegmatis* Encodes Two PhoU Orthologs That Function Redundantly in Signal Transduction

**FIGURE 1:**
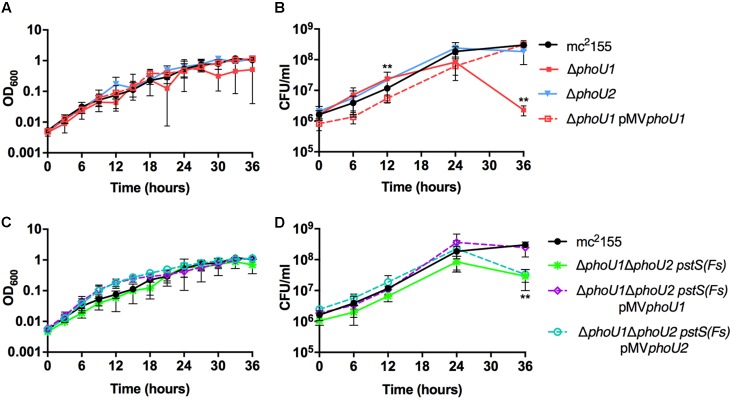
Growth curves of *Mycobacterium smegmatis phoU* deletion mutants. The indicated *M. smegmatis* strains were grown to mid-logarithmic phase and inoculated into complete 7H9 liquid medium at an OD_600_ of 0.01. Cultures were incubated at 37°C with shaking. **(A,C)** Cultures were incubated in a 96 well plate and the OD_600_ was measured hourly (OD_600_ values for every 3 h are shown). **(B,D)** Viable CFU were determined by plating serial dilutions of culture on LB agar. For all panels, results are the mean of three biological replicates ± standard deviations. Asterisks indicate statistically significant differences for Δ*phoU1* **(B)** and Δ*phoU1ΔphoU2 pstS(Fs)* **(D)** compared to the mc^2^155 control: ^∗∗^*P* < 0.005.

**FIGURE 2:**
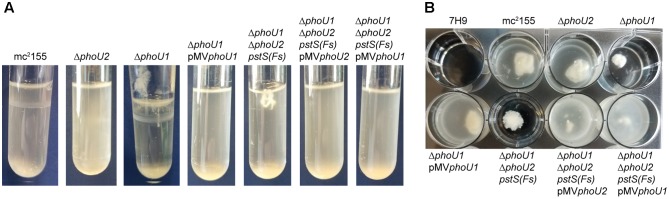
The Δ*phoU1* and Δ*phoU1*Δ*phoU2 pstS(Fs)* mutants have clumping phenotypes in liquid culture. The indicated *M. smegmatis* strains were inoculated into complete 7H9 medium and grown at 37°C with shaking for 72 h in **(A)** tubes or **(B)** a 12-well plate and representative cultures were imaged.

*M. smegmatis* encodes two putative orthologs of *E. coli* PhoU. To determine which of these proteins negatively regulates the *M. smegmatis* SenX3-RegX3 system when P_i_ is abundant, we constructed in-frame unmarked deletions of *phoU1* (*Msmeg_5776*) and *phoU2* (*Msmeg_1605*) by two-step allelic exchange (Upton and McKinney, 2007). The Δ*phoU1* and Δ*phoU2* deletions were confirmed by PCR (data not shown). Growth of each deletion mutant was monitored in complete 7H9 medium, which is P_i_-rich. The Δ*phoU2* mutant exhibited no change in replication rate or overall growth yield compared to wild-type (WT) *M. smegmatis* mc^2^155 in this medium (**Figures 1A,B**). Although the Δ*phoU1* mutant grew at a similar rate as the WT control, it did not reach the same maximal optical density (**Figure 1A**). In addition, the Δ*phoU1* mutant lost viability in stationary phase, with significantly fewer CFU recovered from cultures grown for 36 h as compared to the WT control (**Figure 1B**). The Δ*phoU1* mutant also formed significantly smaller colonies than WT *M. smegmatis* on LB agar plates (**Table 1**). Colonies of the Δ*phoU1* mutant had a sticky phenotype. The Δ*phoU1* mutant also exhibited clumping phenotypes in liquid culture; when grown in tubes it formed a pellicle at the air-liquid interface (**Figure 2A**) and when grown in 12-well plates it formed large visible clumps with fewer dispersed cells compared to the WT control (**Figure 2B**) even in complete 7H9 medium, which contains Tween-80. Clumping may contribute to the reduced growth yield of the Δ*phoU1* mutant. The growth yield, stationary phase viability, colony size and clumping phenotypes of the Δ*phoU1* mutant were all complemented by providing the *phoU1* gene *in trans* on pMV*phoU1* (**Figures 1A,B**, **2** and **Table 1**), confirming that the *phoU1* deletion is responsible for these phenotypes. These phenotypes were also unique to the Δ*phoU1* mutant, as the Δ*phoU2* deletion did not alter colony size (**Table 1**) or clumping in culture (**Figure 2**).

**Table 1:** Colony size of *M. smegmatis* Δ*phoU* mutants.

Strain	Colony area (mm^2^) (mean ± *SD*)^a^	*P*-value vs. mc^2^155
mc^2^155	1.57 ± 0.29	N/A
Δ*phoU1*	0.89 ± 0.15	0.023
Δ*phoU2*	1.43 ± 0.02	0.438
Δ*phoU1* pMV*phoU1*	1.44 ± 0.11	0.506
Δ*phoU1ΔphoU2 pstS(Fs)*	0.93 ± 0.12	0.024
Δ*phoU1ΔphoU2 pstS(Fs)* pMV*phoU1*	1.32 ± 0.10	0.264
Δ*phoU1ΔphoU2 pstS(Fs)* pMV*phoU2*	1.03 ± 0.16	0.038


*^a^The area (mm^*2*^) of 50 individual colonies were determined after 4 days of growth at 37°C on LB agar. Results are the means ± standard deviations of three independent experiments.*

**FIGURE 3:**
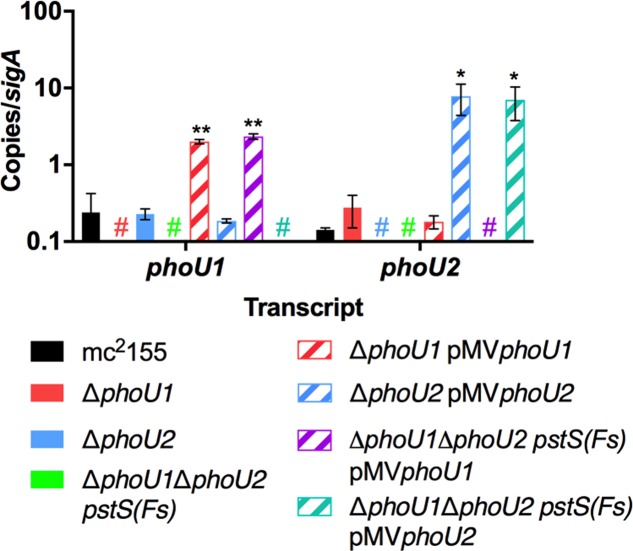
Verification of *phoU* deletion and complemented strains. RNA was extracted from the indicated strains grown in complete 7H9 medium to mid-logarithmic phase. Quantitative RT-PCR was performed to determine abundance of the *phoU1* and *phoU2* transcripts relative to the *sigA* housekeeping control. Results are the mean of three biological replicates ± standard deviations. Asterisks indicate statistically significant differences compared to the mc^2^155 control: ^∗^*P* < 0.05, ^∗∗^*P* < 0.005. Colored # indicates that no transcript was detected in the corresponding deletion mutant.

**FIGURE 4:**
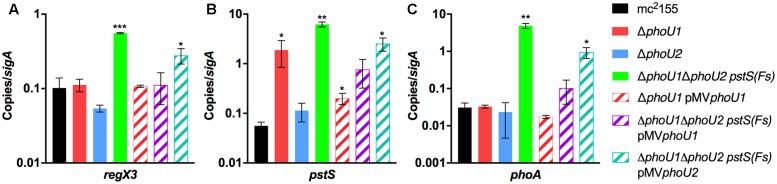
Deletion of both *phoU* orthologs causes aberrant expression of RegX3-regulated genes. RNA was extracted from the indicated *M. smegmatis* strains grown in complete 7H9 medium to mid-logarithmic phase. Quantitative RT-PCR was performed to determine abundance of the *regX3* **(A)**, *pstS* **(B)**, and *phoA* **(C)** transcripts relative to the *sigA* housekeeping control. Results are the mean of three biological replicates ± standard deviations. Asterisks indicate statistically significant differences compared to the mc^2^155 control: ^∗^*P* < 0.05, ^∗∗^*P* < 0.005, ^∗∗∗^*P* < 0.0001.

To determine if either *phoU* single deletion influences activity of SenX3-RegX3, we performed quantitative RT-PCR experiments on RNA extracted from *M. smegmatis* strains grown in P_i_-rich 7H9 medium. We chose 3 known RegX3-dependent genes for analysis: *regX3*, *pstS*, which encodes the substrate-binding protein of the Pst system, and *phoA*, which encodes alkaline phosphatase (Glover et al., 2007). As controls, we tested expression of the *phoU1* and *phoU2* transcripts. The *phoU1* and *phoU2* transcripts were undetectable in the corresponding deletion mutant strains, verifying that the genes were deleted (**Figure 3**). Transcription of *regX3*, *pstS*, and *phoA* was not significantly altered by deletion of *phoU2* (**Figure 4**). Similarly, the Δ*phoU1* mutant showed no significant change in expression of either *regX3* or *phoA* (**Figures 4A,C**). Consistent with these results, alkaline phosphatase activity was unchanged in the Δ*phoU2* mutant and was increased less than twofold in the Δ*phoU1* mutant (**Table 2**). Increased alkaline phosphatase activity of the Δ*phoU1* mutant was not complemented by the pMV*phoU1* plasmid (**Table 2**). In contrast, transcription of *pstS* was significantly increased in the Δ*phoU1* mutant compared to the mc^2^155 control and this phenotype was complemented by pMV*phoU1* (**Figure 4B**), suggesting that the *phoU1* deletion is responsible for this phenotype. Because neither *regX3* nor *phoA* transcription were altered in either the Δ*phoU1* or Δ*phoU2* mutants, our data suggest either that PhoU1 and PhoU2 have partially redundant function in regulating the activity of SenX3-RegX3, or that additional factors contribute to controlling SenX3-RegX3 activity in *M. smegmatis*.

**Table 2:** Alkaline phosphatase activity of *M. smegmatis* Δ*phoU* mutants.

Strain	Alkaline phosphatase units (mean ± *SD*)^a^	*P*-value vs. mc^2^155
mc^2^155	5.24 ± 0.63	
Δ*phoU1*	9.752 ± 0.238	0.0003
Δ*phoU2*	6.24 ± 0.18	0.059
Δ*phoU1* pMV*phoU1*	10.59 ± 1.22	0.003
Δ*phoU1ΔphoU2* pTIC*phoU1* -ATc	4.34 ± 0.23	0.080
Δ*phoU1ΔphoU2* pTIC*phoU1* +ATc	6.47 ± 4.52	0.665
Δ*phoU1ΔphoU2 pstS(Fs)*	44.78 ± 1.80	<0.0001
Δ*phoU1ΔphoU2 pstS(Fs)* pMV*phoU1*	8.71 ± 0.81	0.004
Δ*phoU1ΔphoU2 pstS(Fs)* pMV*phoU2*	13.24 ± 0.65	0.0001


*^a^Alkaline phosphatase units were determined from de-clumped mid-logarithmic phase cultures as described. Results are the means ± standard deviations of 3 independent experiments.*

### The Δ*phoU1* and Δ*phoU2* Mutants Exhibit Decreased Tolerance to Antibiotics Targeting the Cell Wall

**FIGURE 5:**
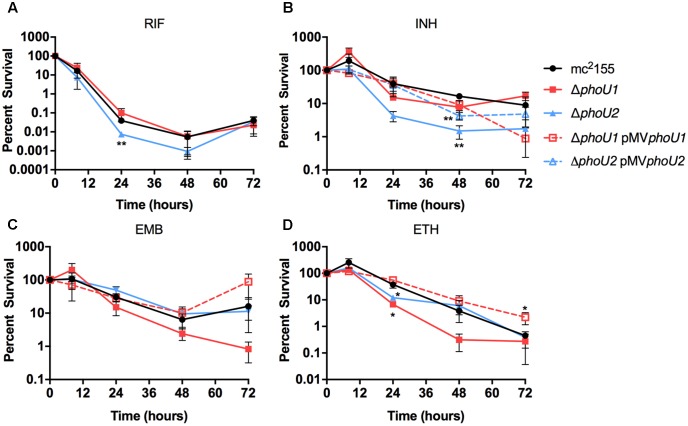
Formation of persister variants by *M. smegmatis phoU* single deletion mutant and complemented strains. The indicated strains were grown to late-logarithmic phase, diluted to OD_600_ of 0.05 in complete 7H9 liquid medium and exposed to the anti-tubercular drug **(A)** rifampicin (RIF, 200 μg/ml), **(B)** isoniazid (INH, 50 μg/ml), **(C)** ethambutol (EMB, 5 μg/ml), or **(D)** ethionamide (ETH, 200 μg/ml) for 72 h. Cultures were incubated at 37°C with aeration and the percent survival was calculated from viable CFU counts determined at 0, 8, 24, 48, and 72 h by plating serially diluted cultures on LB agar medium. Results are the mean of three biological replicates ± standard deviations. Asterisks indicate statistically significant differences compared to the mc^2^155 control: ^∗^*P* < 0.05, ^∗∗^*P* < 0.005.

In *E. coli*, PhoU has previously been implicated as a “persister switch” that is required for formation of antibiotic-tolerant persister variants (Li and Zhang, 2007). Similarly *M. tuberculosis* requires either PhoY1 or PhoY2 to promote persister formation (Namugenyi et al., 2017). To establish if *M. smegmatis* similarly requires PhoU1 or PhoU2 for antibiotic tolerance, we determined the minimal inhibitory concentrations (MICs) of the anti-tubercular drugs rifampicin (RIF), isoniazid (INH), ethambutol (ETB), or ethionamide (ETH) against these mutants. Neither the Δ*phoU1* nor the Δ*phoU2* mutant displayed a substantial change in susceptibility to these drugs (**Table 3**). We also analyzed the rates of death of these mutants following antibiotic treatment to assess persister formation. For the Δ*phoU2* mutant, we observed little change in the kinetics of bacterial death compared to the WT mc^2^155 control during treatment with EMB or ETH (**Figures 5C,D**). The Δ*phoU2* mutant exhibited modestly reduced tolerance to RIF at 24 h compared to the WT control, though this difference was not maintained at later time points (**Figure 5A**). INH killed the Δ*phoU2* mutant more rapidly than mc^2^155, and this phenotype was partially complemented by the pMV*phoU2* plasmid (**Figure 5B**). The Δ*phoU1* mutant was equally susceptible as the mc^2^155 control to RIF (**Figure 5A**). The Δ*phoU1* mutant showed slightly increased susceptibility to INH at 48 h (*P* = 0.035) that was partially complemented by pMV*phoU1* (**Figure 5B**). We also observed modest increases in the death rate of the Δ*phoU1* mutant exposed to either EMB or ETH, though the differences compared to the WT control were significant only for ETH at 24 h (**Figures 5C,D**). These phenotypes were both complemented by pMV*phoU1* (**Figures 5C,D**). These data suggest that PhoU1 and PhoU2 may have independent effects on *M. smegmatis* physiology that influence tolerance to the cell wall targeting antibiotics INH and ETH and to the transcriptional inhibitor RIF.

**Table 3:** Minimal inhibitory concentrations of antibiotics against *M. smegmatis* Δ*phoU* mutants.

Genotype	MIC_90_ (μg/ml)^a^ of:			
	
	RIF	INH	ETB	ETH
WT	3.125	3.125	0.156	25
Δ*phoU1*	3.125	3.125	0.156	25
Δ*phoU2*	3.125	3.125	0.156	12.5
Δ*phoU1ΔphoU2 pstS(Fs)*	0.098	3.125	0.312	12.5
Δ*phoU1ΔphoU2 pstS(Fs)* pMV*phoU1*	1.56	N/A	N/A	N/A
Δ*phoU1ΔphoU2 pstS(Fs)* pMV*phoU2*	1.56–3.125	N/A	N/A	N/A


*^a^MIC_90_ (μg/ml) is the lowest concentration of antibiotic required to inhibit 90% of growth compared to the no drug control. Results are from at least three independent experiments. N/A indicates strain was not tested with this antibiotic. RIF, rifampicin; INH, isoniazid; EMB, ethambutol; ETH, ethionamide.*

### Either PhoU1 or PhoU2 Is Required for *M. smegmatis* Replication *in Vitro*

Our results suggest that PhoU1 and PhoU2 have partially redundant function in negative regulation of SenX3-RegX3 activity in P_i_-rich conditions. To test this idea, we attempted to construct a Δ*phoU1ΔphoU2* double deletion mutant. We introduced the Δ*phoU1* allelic exchange vector into the Δ*phoU2* mutant and obtained 4 independent transformants that contained the vector integrated via the cloned regions of homology either upstream or downstream of the *phoU1* gene. After counter-selection against the allelic exchange vector by plating on sucrose, we screened a total of 172 sucrose resistant, kanamycin sensitive (Kan^S^), hygromycin sensitive (Hyg^S^) colonies for the Δ*phoU1* deletion by PCR. None of these isolates harbored the Δ*phoU1* deletion. These data suggested that *M. smegmatis* requires either PhoU1 or PhoU2 for viability under the growth conditions used.

**FIGURE 6:**
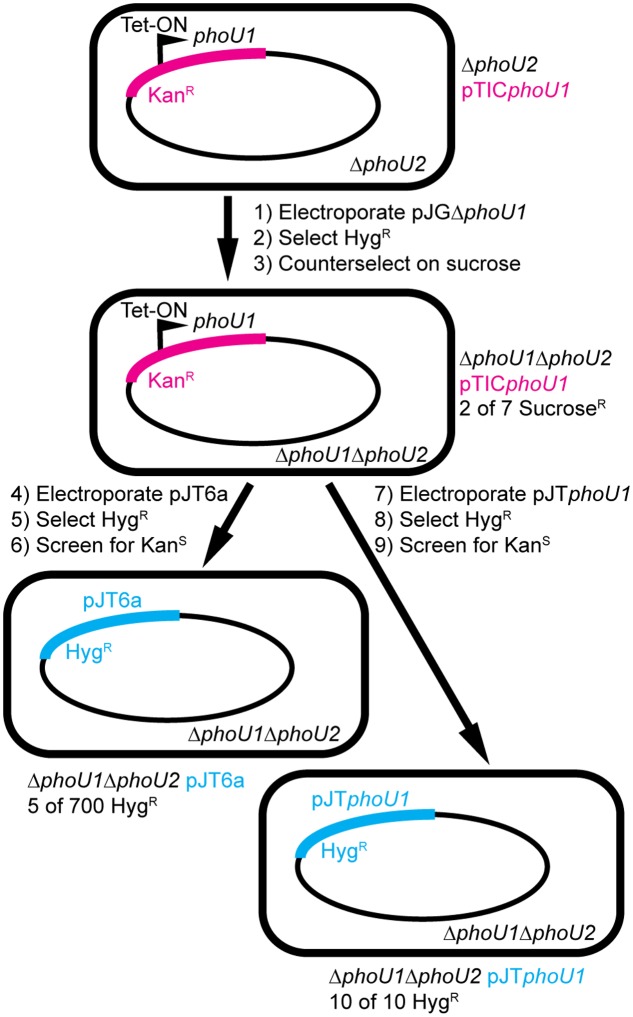
Construction of *M. smegmatis* Δ*phoU1ΔphoU2* double deletion mutants by a plasmid swapping method. The *phoU1* and *phoU2* genes were deleted from the *M. smegmatis* chromosome by allelic exchange in a background containing a tetracycline-inducible copy of *phoU1* provided *in trans* on the integrative plasmid pTIC*phoU1* that confers kanamycin (Kan) resistance (steps 1–3). The pTIC*phoU1* plasmid was then removed by swapping with either the empty non-compatible integrative plasmid pJT6a (steps 4–6) or pJT*phoU1* (steps 7–9), which confer hygromycin (Hyg) resistance. The frequencies with which strains of the correct genotype were isolated are indicated.

To determine if *phoU1* and *phoU2* are jointly essential for *M. smegmatis* viability, we attempted to delete *phoU1* in the Δ*phoU2* background with a complementing copy of *phoU1* provided *in trans*. We cloned *phoU1* under the control of a tetracycline-inducible promoter on the integrating plasmid pTIC10a to enable conditional *phoU1* expression. We created a Δ*phoU2* pTIC*phoU1* strain and then electroporated this strain with the pJGΔ*phoU1* allelic exchange vector (**Figure 6**, steps 1–2). We obtained transformants in which the allelic exchange vector was integrated via either the upstream or downstream region of homology. After counter-selection on sucrose, we screened a total of 7 sucrose resistant, Hyg^S^ colonies for the Δ*phoU1* deletion by PCR. Two of these isolates had the Δ*phoU1* deletion (**Figure 6**, step 3). The ease with which we constructed the Δ*phoU1ΔphoU2* double mutant when a complementing copy of one gene was provided *in trans* suggests that *M. smegmatis* requires *phoU1* or *phoU2* for *in vitro* replication. The Δ*phoU1ΔphoU2* pTIC*phoU1* strain exhibited alkaline phosphate activity similar to the WT control, even in the absence of induction with anhydrotetracycline (ATc) (**Table 2**), suggesting that leaky expression of *phoU1* from the pTIC*phoU1* plasmid was sufficient for negative regulation of SenX3-RegX3. In fact, *phoU1* was expressed at a significantly higher level from the pTIC*phoU1* plasmid even in the absence of ATc compared to the mc^2^155 control (Supplementary Figure S1).

To confirm that at least one of the *phoU* genes is essential for *M. smegmatis* viability, we attempted to swap the pTIC*phoU1* plasmid with a non-compatible plasmid containing a Hyg^R^ marker, pJT6a (Rosen et al., 2017). The Δ*phoU1ΔphoU2* pTIC*phoU1* strain was electroporated with pJT6a and transformants were selected on LB containing Hyg (**Figure 6**, steps 4–5). *M. smegmatis* mc^2^155 containing pTIC*phoU1* was similarly electroporated with pJT6a as a control. We obtained 4500 CFU/ml Hyg^R^ transformants from the WT pTIC*phoU1* control, but only 150 CFU/ml Hyg^R^ transformants from the Δ*phoU1ΔphoU2* pTIC*phoU1* strain, suggesting that there is strong selective pressure against loss of the pTIC*phoU1* plasmid in the Δ*phoU1ΔphoU2* background. The Hyg^R^ transformants were then screened for loss of the Kan^R^ marker on pTIC*phoU1* (**Figure 6**, step 6). For the WT control, all 10 Hyg^R^ colonies screened were sensitive to Kan, suggesting that they had lost the pTIC*phoU1* plasmid. PCR analysis on 8 of these Hyg^R^ Kan^S^ isolates confirmed that they all contained the pJT6a plasmid and not pTIC*phoU1* (data not shown). In contrast, for the Δ*phoU1ΔphoU2* pTIC*phoU1* strain, of the 700 Hyg^R^ transformants that were screened, only 75 were sensitive to Kan, suggesting that the majority had not lost the pTIC*phoU1* plasmid. PCR analysis indicated that 63 of the Hyg^R^ Kan^S^ isolates still harbored the pTIC*phoU1* plasmid and did not contain pJT6a, suggesting that some recombination event had occurred to swap the antibiotic resistance markers in these strains. Using PCR, we ultimately identified only five Hyg^R^ Kan^S^ isolates that had successfully undergone the plasmid swap (**Figure 6** and data not shown). All five of these isolates were also confirmed to have both the Δ*phoU1* and Δ*phoU2* deletions by PCR (data not shown).

To determine if the plasmid swap was inefficient in the Δ*phoU1ΔphoU2* pTIC*phoU1* strain due to some deficiency in recombination as opposed to the loss of both *phoU* genes, we performed a similar plasmid swapping experiment using pJT*phoU1* (**Figure 6**, steps 7–9). We observed a substantial increase in the number of transformants obtained with the pJT*phoU1* plasmid compared to the empty pJT6a plasmid. We obtained 5800 CFU/ml Hyg^R^ transformants from the WT mc^2^155 pTIC*phoU1* background and 4200 CFU/ml Hyg^R^ transformants from the Δ*phoU1ΔphoU2* pTIC*phoU1* strain. In addition, all 10 of the Hyg^R^ transformants that we screened on each strain background were sensitive to Kan, suggesting that they had replaced pTIC*phoU1* with pJT*phoU1* (**Figure 6**). Taken together, these data suggest that *M. smegmatis* requires either *phoU1* or *phoU2* for viability under the *in vitro* culture conditions that we used and that the five Δ*phoU1ΔphoU2* pJT6a strains we generated may harbor secondary suppressor mutations that enable their growth.

### Whole-Genome Sequencing Identifies Independent Suppressor Mutations in Genes Encoding the Pst Phosphate Transporter in the Δ*phoU1ΔphoU2* Mutants

To determine if the Δ*phoU1ΔphoU2* mutants that we isolated by the plasmid swap method contain suppressor mutations that enable their *in vitro* replication, we performed whole-genome sequencing on all five of the Δ*phoU1ΔphoU2* pJT6a isolates. We compared the sequences of these strains to the sequence of our WT *M. smegmatis* mc^2^155 control, and identified two mutations in each strain. All five Δ*phoU1ΔphoU2* pJT6a isolates harbored a G818T substitution in *Msmeg_1387*, which encodes a putative acyl-CoA dehydrogenase, resulting in a S273I transition in the Msmeg_1387 protein. We confirmed the G818T substitution in each Δ*phoU1ΔphoU2* pJT6a isolate by PCR and sequencing, and determined that it was also present in the Δ*phoU1ΔphoU2* pTIC*phoU1* parent strain, suggesting that this single nucleotide polymorphism (SNP) is not responsible for suppressing the essentiality of the *phoU* genes. Each Δ*phoU1ΔphoU2* pJT6a strain also harbored an independent mutation in *pstS*, *pstC* or *pstB*, which encode components of the Pst P_i_ transporter (**Table 4**). These mutations were likewise confirmed by PCR and sequencing. Four of the strains (#504, 521, 625, and 664) contained SNPs that were predicted to severely disrupt the function of the encoded protein by causing a frame shift. The mutation in strain #518 was predicted to introduce a single glutamine residue into a region of PstB that contains primarily polar amino acids. This mutation may disrupt interaction of the PstB ATPase with other components of the Pst system. These data suggest that in the absence of PhoU1 and PhoU2, P_i_ import by the Pst system is toxic. All experiments described below used the Δ*phoU1ΔphoU2* pJT6a #504 mutant, which harbors a frameshift mutation in *pstS* that is predicted to truncate more than 70% of the PstS protein (**Table 4**). Hereafter, we refer to this strain as the Δ*phoU1ΔphoU2 pstS(Fs)* triple mutant.

**Table 4:** Mutations in genes encoding Pst system components identified by whole-genome sequencing of five Δ*phoU1ΔphoU2* pJT6a isolates.

Strain	Position (bp)^a^	Gene	Mutation^b^	NT_Pos^c^	AA_Pos^d^	Effect
Δ*phoU1ΔphoU2* pJT6a #504	5,855,570	*pstS*	G insertion, frameshift variant	254/1,137	85/378	Alters 28 AA; truncates PstS from 378 to 112 AA
Δ*phoU1ΔphoU2* pJT6a #518	5,852,157-5,852,159	*pstB*	AGC insertion	465/777	155/258	Adds Gln; alters polar region of PstB
Δ*phoU1ΔphoU2* pJT6a #521	5,852,274	*pstB*	C insertion, frameshift variant	453/777	151/258	Alters 107 AA; elongates PstB by 68 AA
Δ*phoU1ΔphoU2* pJT6a #625	5,855,784	*pstS*	G deletion, frameshift variant	135/1,137	45/378	Alters 14 AA; truncates PstS from 378 to 59 AA
Δ*phoU1ΔphoU2* pJT6a #664	5,854,548	*pstC*	G insertion, frameshift variant	162/1,057	54/351	Alters 172 AA; truncates PstC from 351 to 226 AA


*^a^Position on the *M. smegmatis* mc^*2*^155 chromosome. ^b^All mutation descriptions refer to the base(s) altered on the coding strand. ^c^Nucleotide position of the mutation relative to WT gene length. ^d^Amino acid position of the mutation relative to the WT protein.*

### The *M. smegmatis* Δ*phoU1ΔphoU2 pstS(Fs)* Mutant Has an *in Vitro* Growth Defect

To quantitatively assess the requirement of *phoU1* and *phoU2* for *M. smegmatis* growth *in vitro*, we performed growth curves. Like the Δ*phoU1* mutant, the Δ*phoU1 ΔphoU2 pstS(Fs)* mutant grew at a similar rate as WT *M. smegmatis* in liquid medium *in vitro* (**Figure 1C**), but formed significantly smaller colonies on LB agar plates (**Table 1**). Colonies of the Δ*phoU1ΔphoU2 pstS(Fs)* mutant typically did not appear until after 3–4 days of incubation at 37°C while the WT and single deletion mutant colonies were visible after only 2 days. This growth defect on plates was complemented by pMV*phoU1* (**Table 1**). The Δ*phoU1ΔphoU2 pstS(Fs)* mutant also lost viability in stationary phase, though this phenotype was less pronounced as compared to the Δ*phoU1* single mutant (**Figure 1D**). Only *phoU1* provided *in trans* could complement this stationary phase survival phenotype (**Figure 1D**). Finally, the Δ*phoU1ΔphoU2 pstS(Fs)* mutant produced a pellicle at the air-liquid interface of cultures grown in tubes (**Figure 2A**), and aggregated in complete 7H9 liquid medium similar to the Δ*phoU1* mutant (**Figure 2B**). These clumping phenotypes were both complemented by providing either *phoU1* or *phoU2 in trans* (**Figure 2**). These data suggest that *M. smegmatis* requires either *phoU1* or *phoU2* for normal replication in culture.

### *M. smegmatis* PhoU1 and PhoU2 Have Overlapping Functions in Inhibition of SenX3-RegX3 Activation

To test if PhoU1 and PhoU2 function redundantly to regulate activity of SenX3-RegX3, we analyzed expression of known RegX3-regulated genes in the Δ*phoU1ΔphoU2 pstS(Fs)* mutant. By quantitative RT-PCR, the *phoU1* and *phoU2* transcripts were undetectable in this mutant (**Figure 3**), confirming that both *phoU* genes were deleted. Expression of *regX3*, *pstS*, and *phoA* was significantly increased 5-fold, 110-fold, and 158-fold, respectively, in the Δ*phoU1ΔphoU2 pstS(Fs)* triple mutant compared to the WT mc^2^155 control (**Figure 4**). Expression of *pstS* was also significantly increased in the Δ*phoU1ΔphoU2 pstS(Fs)* mutant relative to the Δ*phoU1* single mutant (*P* = 0.004). We observed similar increases in the abundance of the *regX3*, *pstS*, and *phoA* transcripts in the #518 and #521 Δ*phoU1ΔphoU2* pJT6a strains that each harbor an independent compensatory mutation in *pstB* (data not shown). We attempted to complement the gene expression phenotypes of the Δ*phoU1ΔphoU2 pstS(Fs)* mutant with either *phoU1* or *phoU2*. The *phoU1* and *phoU2* transcripts were significantly elevated in the respective complemented strains compared to the WT mc^2^155 control (**Figure 3**). Complementation of the Δ*phoU1ΔphoU2 pstS(Fs)* mutant with pMV*phoU1* restored expression of the *regX3* and *phoA* transcripts to levels that were not significantly different from the WT mc^2^155 control (**Figures 4A,C**). Complementation with pMV*phoU1* also reduced transcription of *pstS* to a level that was only 13-fold higher and not significantly different from the WT mc^2^155 control (**Figure 4B**). Complementation with pMV*phoU2* only partially reversed the over-expression of the *regX3*, *pstS*, or *phoA* transcripts; levels of these transcripts were reduced compared to the Δ*phoU1ΔphoU2 pstS(Fs)* mutant but remained significantly elevated relative to the WT control (**Figure 4**). Consistent with these results, we observed a 8.5-fold increase in alkaline phosphatase activity in the Δ*phoU1ΔphoU2 pstS(Fs)* strain that was complemented by providing either *phoU1* or *phoU2 in trans*, though full complementation was only observed with pMV*phoU1* (**Table 2**). These data suggest that the PhoU1 and PhoU2 proteins have overlapping functions in negatively regulating SenX3-RegX3 activity in P_i_-rich growth conditions. They further suggest that PhoU1 plays a more dominant role in fulfilling this P_i_ sensing signal transduction function. Since the gene expression phenotypes of the Δ*phoU1ΔphoU2 pstS(Fs)* mutant were complemented with pMV*phoU1*, our data also suggest that deletion of both *phoU* genes, not the *pstS* frameshift mutation in this strain, is responsible for activation of SenX3-RegX3.

### The *M. smegmatis* Δ*phoU1ΔphoU2 pstS(Fs)* Mutant Is Hyper-Susceptible to RIF and Forms Persisters with Reduced Frequency

**FIGURE 7:**
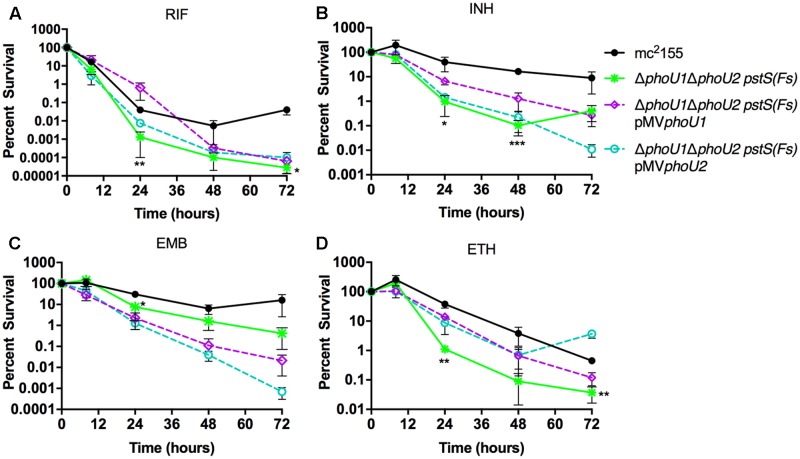
Formation of persister variants by *M. smegmatis ΔphoU1ΔphoU2 pstS(Fs)* mutant and complemented strains. The indicated strains were grown to late-logarithmic phase, diluted to OD_600_ of 0.05 in complete 7H9 liquid medium and exposed to the anti-tubercular drug **(A)** rifampicin (RIF, 200 μg/ml), **(B)** isoniazid (INH, 50 μg/ml), **(C)** ethambutol (EMB, 5 μg/ml), or **(D)** ethionamide (ETH, 200 μg/ml) for 72 h. Cultures were incubated at 37°C with aeration and the percent survival was calculated from viable CFU counts determined at 0, 8, 24, 48, and 72 h by plating serially diluted cultures on LB agar medium. Results are the mean of three biological replicates ± standard deviations. Asterisks indicate statistically significant differences compared to the mc^2^155 control: ^∗^*P* < 0.05, ^∗∗^*P* < 0.005, ^∗∗∗^*P* < 0.0001.

To determine if the *M. smegmatis* PhoU proteins are jointly required for antibiotic tolerance, similar to the *M. tuberculosis* PhoY proteins, we performed MIC and persister assays on the Δ*phoU1ΔphoU2 pstS(Fs)* mutant. MICs of the Δ*phoU1ΔphoU2 pstS(Fs)* mutant for INH, EMB and ETH, were unchanged relative to the WT control, but the MIC for RIF was reduced 32-fold (**Table 3**). Complementation with either *phoU1* or *phoU2* restored the RIF MIC to the WT level (**Table 3**). These data suggest that constitutive activation of SenX3-RegX3 contributes to increased susceptibility to RIF. The Δ*phoU1ΔphoU2 pstS(Fs)* mutant was also killed more rapidly by RIF, INH and ETH in liquid culture compared to the WT mc^2^155 control, suggesting that it forms fewer persister variants (**Figures 7A,B,D**). The Δ*phoU1ΔphoU2 pstS(Fs)* mutant also showed a trend toward decreased tolerance to EMB, though it was not statistically significant (**Figure 7C**). However, in most cases these phenotypes were only partially reversed by complementation with either *phoU1* or *phoU2*. For RIF, complementation with pMV*phoU1* restored tolerance at early time points, but failed to prevent the increased killing observed at later time points (**Figure 7A**). For INH, complementation with pMV*phoU1* resulted in an intermediate antibiotic tolerance phenotype (**Figure 7B**), consistent with the fact that the Δ*phoU2* single mutant exhibited a modest defect in survival of INH treatment (**Figure 5B**). Complementation with either pMV*phoU1* or pMV*phoU2* similarly led to intermediate tolerance to ETH (**Figure 7D**), consistent with the fact that the Δ*phoU1* mutant was also more susceptible to this drug (**Figure 5D**). In contrast, both complemented strains exhibited further enhanced susceptibility to EMB (**Figure 7C**). It is possible that over-expression of *phoU1* or *phoU2* from the episomal plasmid is detrimental and prevents complete complementation of these antibiotic tolerance phenotypes. Alternatively, it is possible that the point mutation in *pstS*, which is predicted to disrupt P_i_ transport by the Pst system, is partially responsible for the decreased antibiotic tolerance.

### The Δ*phoU1ΔphoU2 pstS(Fs)* Mutant Accumulates Polyphosphate

Mutation of *phoU* has previously been reported to cause accumulation of polyphosphate (polyP) a linear polymer of phosphate residues linked by high-energy phosphoanhydride bonds (Kornberg et al., 1999), in a variety of bacterial species including *M. marinum* and *M. tuberculosis* (Morohoshi et al., 2002; Wang et al., 2013; Namugenyi et al., 2017). We therefore examined whether deletion of the *M. smegmatis phoU* genes altered polyP storage during growth in P_i_-rich 7H9 medium. The Δ*phoU2* mutant showed no significant alteration in polyP accumulation (**Table 5**). Intracellular polyP was increased approximately twofold in the Δ*phoU1* mutant though this difference was not statistically significant and this phenotype was not complemented with the pMV*phoU1* plasmid (**Table 5**). In contrast, the Δ*phoU1ΔphoU2 pstS(Fs)* triple mutant exhibited a statistically significant 10-fold increase in polyP storage relative to the mc^2^155 control (**Table 5**). Complementation with either *phoU1* or *phoU2* resulted in only a partial reduction in polyP accumulation, though pMV*phoU1* complemented the phenotype more effectively than pMV*phoU2* (**Table 5**). These data suggest that the *M. smegmatis* PhoU proteins regulate polyP storage. Since the Δ*phoU1ΔphoU2 pstS(Fs)* mutant accumulates polyP despite the mutation in *pstS*, our data further suggest that P_i_ uptake through the Pst system does not contribute to increased polyP storage.

**Table 5:** Polyphosphate quantification in *M. smegmatis* Δ*phoU* mutants.

Strain	nmol polyP/mg total protein (mean ± *SD*)^a^	*P*-value vs. mc^2^155
mc^2^155	0.020 ± 0.002	
Δ*phoU1*	0.038 ± 0.014	0.094
Δ*phoU2*	0.018 ± 0.002	0.188
Δ*phoU1* pMV*phoU1*	0.040 ± 0.014	0.073
Δ*phoU1ΔphoU2 pstS(Fs)*	0.206 ± 0.048	0.0022
Δ*phoU1ΔphoU2 pstS(Fs)* pMV*phoU1*	0.043 ± 0.004	0.047
Δ*phoU1ΔphoU2 pstS(Fs)* pMV*phoU2*	0.061 ± 0.025	0.0015


*^a^Polyphosphate was extracted from mid-logarithmic phase cultures. Results are mean values ± standard deviations of 3 independent experiments.*

## Discussion

Although P_i_ limitation activates the *M. smegmatis* two-component signal transduction system SenX3-RegX3 (Glover et al., 2007), the molecular mechanisms mediating P_i_ sensing in this organism were not previously described. Here we demonstrate that the two *M. smegmatis* PhoU orthologs exhibit partially redundant functions in controlling activation of SenX3-RegX3 and that PhoU function is essential for *M. smegmatis* replication *in vitro*. Only deletion of both *phoU1* and *phoU2* led to constitutive expression of all three known RegX3-regulated genes. Each of the Δ*phoU1ΔphoU2* double mutant strains that we isolated harbored independent compensatory mutations in genes encoding the Pst P_i_ transporter, which alleviated the essentiality of the two PhoU proteins. Our data are consistent with studies in other bacteria that implicated the Pst P_i_ transporter in mediating the growth defect or lethality associated with *phoU* inactivation (Hirota et al., 2013; Lubin et al., 2016; diCenzo et al., 2017). Mutations disrupting the *M. smegmatis* Pst transporter also constitutively activate SenX3-RegX3 (Kriakov et al., 2003; Glover et al., 2007). However, our data suggest that the deletion of both *phoU* genes causes constitutive expression of the RegX3 regulon in the Δ*phoU1ΔphoU2 pstS(Fs)* mutant independent of the *pstS* mutation because we could complement the gene expression phenotypes with *phoU1* provided *in trans.* Our data therefore suggest that PhoU1 can directly regulate SenX3-RegX3 activity, independent of the *pstS* mutation, and that PhoU1 plays a more dominant role in P_i_ signaling than PhoU2. Furthermore, our data suggest that the *M. smegmatis* PhoU proteins function redundantly to regulate Pst P_i_ transport activity, since in their absence a functional Pst system is toxic.

Our results in *M. smegmatis* correspond to our previous report that the *M. tuberculosis* PhoU orthologs (PhoY1 and PhoY2) function redundantly to regulate SenX3-RegX3 activity (Namugenyi et al., 2017). However, we were able to generate the *M. tuberculosis* Δ*phoY1ΔphoY2* double mutant using standard gene deletion methods, suggesting differences in the function of the PhoU orthologs between *M. smegmatis* and *M. tuberculosis*. It is possible that the *M. tuberculosis* PhoY proteins do not regulate P_i_ transport by the Pst system or that the *M. tuberculosis* Pst system transports P_i_ at a slower rate that does not cause toxic P_i_ accumulation. Further work will be required to conclusively establish the role of the *M. smegmatis* PhoU proteins or the *M. tuberculosis* PhoY proteins in regulating P_i_ uptake by the Pst system.

The *M. smegmatis phoU* mutants also exhibited reduced tolerance to several anti-tubercular drugs. In particular, the Δ*phoU1ΔphoU2 pstS(Fs)* mutant was markedly more susceptible to RIF, a phenotype we previously observed for the *M. tuberculosis* Δ*phoY1ΔphoY2* mutant (Namugenyi et al., 2017). In *M. tuberculosis*, we demonstrated that constitutive activation of RegX3 caused RIF susceptibility (Namugenyi et al., 2017), so we predict that RegX3 activation is similarly responsible for RIF susceptibility in *M. smegmatis*. However, RegX3 is essential for growth of *M. smegmatis in vitro* (James et al., 2012), so we were unable to generate a *regX3* deletion mutant to directly test this prediction. Our future studies will include performing transposon mutagenesis screens to identify factors that contribute to the enhanced RIF susceptibility of the Δ*phoU1ΔphoU2 pstS(Fs)* mutant.

The *M. smegmatis phoU* single and double deletion mutants also exhibited decreased phenotypic tolerance to the cell wall targeting antibiotics INH, EMB and ETH, revealed by examination of the death kinetics during antibiotic treatment. In general the Δ*phoU1ΔphoU2 pstS(Fs)* mutant was the most susceptible, but the Δ*phoU1* mutant also exhibited reduced tolerance to ETH and EMB. Susceptibility to these drugs and the visible clumping phenotype of the Δ*phoU1* mutant in liquid culture may both be caused by alterations to cell wall structure. In *M. smegmatis*, the Pst system has previously been implicated in regulating production of the alpha-glucan capsule (van de Weerd et al., 2016). However, we found no evidence for differential expression of genes involved in alpha-glucan synthesis in any of our Δ*phoU* mutants (data not shown) suggesting that other cell wall alterations are responsible for these phenotypes. Further study of changes to the cell wall in the Δ*phoU1* mutant and determining whether these changes correlate with antibiotic susceptibility will be important as it could reveal new molecular targets that would enhance the activity of existing drugs.

The *M. smegmatis* Δ*phoU1ΔphoU2 pstS(Fs)* mutant also accumulated polyP, similar to the *M. tuberculosis* Δ*phoY1ΔphoY2* mutant (Namugenyi et al., 2017), despite harboring a mutation predicted to render the PstS substrate binding domain of the Pst P_i_ transporter non-functional. Our data contrast with evidence that polyP accumulation by *phoU* mutants in *E. coli, Pseudomonas aeruginosa*, and *Sinorhizobium meliloti* requires a functional Pst system (Morohoshi et al., 2002; Hirota et al., 2013; diCenzo et al., 2017; Peng et al., 2017). It is possible that polyP would accumulate to an even higher level in a *M. smegmatis phoU* double mutant with a fully functional Pst system. Nevertheless, our data suggest either that P_i_ uptake via other transporters is dys-regulated in the *M. smegmatis* Δ*phoU1ΔphoU2 pstS(Fs)* mutant leading to increased polyP storage or that the *M. smegmatis* PhoU proteins directly modulate polyP accumulation by regulating expression or activity of enzymes that synthesize or degrade polyP. In addition to the Pst system, *M. smegmatis* has at least two other high-affinity P_i_ transporters, the Phn system and at least one other that remains to be identified (Gebhard et al., 2006). The PhoU proteins may regulate expression and/or activity of these alternative P_i_ transporters to influence polyP storage.

Although the *M. smegmatis* Δ*phoU1* mutant did not constitutively express all genes in the RegX3 regulon, it exhibited several unique phenotypes including stickiness of colonies, clumping in liquid culture, reduced viability in stationary phase and over-expression of *pstS*. Each of these phenotypes could be complemented suggesting that they are caused by the *phoU1* deletion. Furthermore, these phenotypes, particularly reduced viability in stationary phase, were more pronounced in the Δ*phoU1* mutant compared to the Δ*phoU1ΔphoU2 pstS(Fs)* triple mutant. It is possible that the frameshift mutation in *pstS* partially alleviates the loss of *phoU1* in the triple mutant strain. Our data suggest that *M. smegmatis* PhoU1 has one or more functions that are independent and distinct from PhoU2, which may include more robust regulation of Pst P_i_ transport activity. PhoU1 is encoded adjacent to the *pstSCAB* operon, suggesting there could be specificity conferred by co-evolution of the *phoU1* and *pstSCAB* genes or a requirement for these genes to be close proximity to enable their co-regulation. Over-expression of *pstS* by the Δ*phoU1* mutant suggests that PhoU1 influences activity of a transcriptional regulator that controls expression of the *pst* operon. RegX3 may be weakly activated in the Δ*phoU1* mutant, leading to expression of genes with high affinity binding sites for the phosphorylated form of RegX3, which could include the *pst* genes. Alternatively, PhoU1 may control the activation of other transcriptional regulators. Gene expression profiling of the Δ*phoU1* mutant could provide further insight into the basis for its *in vitro* growth phenotypes and clues to the transcriptional regulatory pathways that it influences.

Overall, our data suggest that both PhoU1 and PhoU2 participate in the P_i_ sensing system that controls activation of SenX3-RegX3 and expression of the P_i_ starvation RegX3 regulon in *M. smegmatis*. The PhoU1 and PhoU2 proteins exhibit 82% sequence similarity, so both proteins likely contain conserved domains that enable interactions with SenX3 and the Pst system. Further study of the interactions among these proteins could reveal the molecular basis for P_i_ sensing. Our work also suggests that *M. smegmatis* can serve as a model organism to discover compounds that inhibit PhoU1 and PhoU2 function to constitutively activate RegX3. Such compounds would be predicted to enhance susceptibility of mycobacteria to existing anti-tubercular drugs, including RIF.

## Author Contributions

AT conceived the project and designed the strategy. AB, BE, MM, and JB carried out the experiments. AB and AT analyzed the data. AB and AT wrote the manuscript.

## Conflict of Interest Statement

The authors declare that the research was conducted in the absence of any commercial or financial relationships that could be construed as a potential conflict of interest.

## Footnotes

1 https://www.geneious.com
