# Supplementary material for: Mycobacterium smegmatis PhoU Proteins Have Overlapping Functions in Phosphate Signaling and Are Essential
Source: Front Microbiol. 2017 Dec 18;8:2523. doi: 10.3389/fmicb.2017.02523 (PMC5741670; doi:10.3389/fmicb.2017.02523)
Supplement: Supplementary file 1 [file Image_1.PDF]

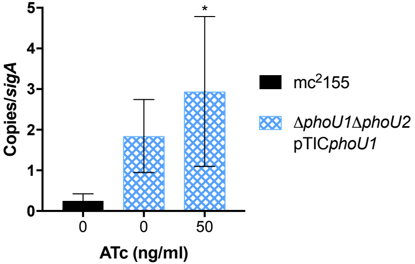

FIG S1. pTIC10a-*phoU1* leaky expression. RNA was extracted from the indicated strains grown to mid-logarithmic phase in complete 7H9 medium supplemented with the indicated anhydrotetracycline (ATc) concentration. Quantitative RT-PCR was performed to determine abundance of the *phoU1* transcripts relative to the sigA housekeeping control. Results are the mean of three biological replicates ± standard deviations. Asterisks indicate statistically significant differences compared to the mc^2155 control: * $P < 0.05$.

Table S1. Plasmids used in this study.

Plasmid	Genotype	Reference
pCR2.1 TOPO	PCR cloning vector Amp^R Kan^R	Invitrogen
pJG1100	Allelic exchange suicide vector Kan^R Hyg^R *sacB*	(1)
pBE101	pJG1100::Δ*phoU1* (*Msmeg_5776*), Kan^R, Hyg^R, *sacB*	This work
pBE102	pJG1100::Δ*phoU2* (*Msmeg_1605*), Kan^R, Hyg^R, *sacB*	This work
pMV261	Episomal vector with *hsp60* promoter Kan^R	(2)
pMV*phoU1*	pMV261::*phoU1*, Kan^R	This work
pMV*phoU2*	pMV261::*phoU2*, Kan^R	This work
pTIC10a	Integrating vector with codon-optimized TetR, P_{smyc}-TetO, Kan^R	(3)
pTIC*phoU1*	pTIC10a::*phoU1*, Kan^R	This work
pJT6a	Integrating vector with codon-optimized TetR, P_{smyc}-TetO, Hyg^R	(4)
pJT*phoU1*	pJT6a::*phoU1*, Hyg^R	This work

1. **Kirksey MA, Tischler AD, Siméone R, Hisert KB, Uplekar S, Guilhot C, McKinney JD.** 2011. Spontaneous phthiocerol dimycocerosate-deficient variants of *Mycobacterium tuberculosis* are susceptible to gamma interferon-mediated immunity. Infect Immun **79:**2829-2838.
2. **Stover CK, de la Cruz VF, Fuerst TR, Burlein JE, Benson LA, Bennett LT, Bansal GP, Young JF, Lee MH, Hatfull GF, Snapper SB, Barletta RG, Jacobs WR, Jr., Bloom BR.** 1991. New use of BCG for recombinant vaccines. Nature **351:**456-460.
3. **Glover RT, Kriakov J, Garforth SJ, Baughn AD, Jacobs WRJ.** 2007. The two-component regulatory system *senX3-regX3* regulates phosphate-dependent gene expression in *Mycobacterium smegmatis*. J Bacteriol **189:**5495-5503.
4. **Rosen BC, Dillon NA, Peterson ND, Minato Y, Baughn AD.** 2017. Long-chain fatty acyl coenzyme A ligase FadD2 mediates intrinsic pyrazinamide resistance in *Mycobacterium tuberculosis*. Antimicrob Agents Chemother **61:**e02130-16.

Table S2. Oligonucleotide primers used for cloning or strain construction in this study.

Name	Purpose	Sequence (5'-3')[a]
1605F1	Upstream Δ*phoU2*	ATGCTTAATTAAACACTGCGCCTGCTCAAC
1605R1	Upstream Δ*phoU2*	ATGCCCTAGGAGCTCG**CAT**CGCCCATGAC
1605F2	Downstream Δ*phoU2*	GCATCCTAGGCTCGCC**TGA**TTACCGGCTAGC
1605R2	Downstream Δ*phoU2*	ATGCGGCGCGCCCGTTGATGCGCTCTGCGAACT
5776F1	Upstream Δ*phoU1*	GCATTTAATTAACCGCATCACGTTCGTCACCAT
5776R1	Upstream Δ*phoU1*	ATGCCCTAGGATGGTACTGGATCCG**CAT**GCA
5776F2	Downstream Δ*phoU1*	GCATCCTAGGAAGGTCACCACGCAGCAG
5776R2	Downstream Δ*phoU1*	ATGCGGCGCGCCCCCTTGGTGAGGTTGGTGAG
1605F3	Check Δ*phoU2*	GGAAACAGCTGCTGCGCAAC
1605R3	Check Δ*phoU2*	CGAGCTTTTCGGCAAACTCGA
1605F4	Check Δ*phoU2*	GGATGGGTACCTACGTCCTCA
1605R4	Check Δ*phoU2*	CCTGGTGATCTGCTCCGTAAC
5776F3	Check Δ*phoU1*	ACCAAGGATCTCGTGGACCTC
5776R3	Check Δ*phoU1*	CGACGGGAAGCTCGATCTCCT
5776F4	Check Δ*phoU1*	GGTGTTCACCGCCTAATCTGG
5776R4	Check Δ*phoU1*	CGCGACTTCCTGATGCGCTA
1605CF	pMV*phoU2* cloning	ATGAATTCGGTACCTACGTCCTCATCCGG
1605CR	pMV*phoU2* cloning	ATAAGCTTCTGGTGATCTGCTCCGTACC
5776CF	pMV*phoU1* cloning	ATGAATTCCACCAGCAGCTTCGTGACTG
5776CR	pMV*phoU1* cloning	ATAAGCTTCCGATCAGCCGTAGGTCA
TIC5776F	pTIC10a-*phoU1* cloning	ATAAGCTTCACCAGCAGCTTCGTGACTG
TIC5776R	pTIC10a-*phoU1* cloning	ATGAATTCCCGATCAGCCGTAGGTCA
pTfor	Check plasmid switch	CATCCCGGCGTTGATCTGTG
pTIC6a_R	Check plasmid switch	TTTTCTTAAGGAGCAAGACGTTTCCCGTT
1387F2	Verify point mutation	TGCGACATCTTCCTGGTGC
1387R2	Verify point mutation	AGCACGTTGCGCATCAGC
DM504_pstSF	Verify point mutation	ATGAGCGGCGAATACGTTGC
DM504_pstSR	Verify point mutation	GGGAACCTGTCGGTCATGTG
DM518_pstBF	Verify point mutation	CGCTCACGCTCATCCTGC

DM518_pstBR	Verify point mutation	GTCCATGGTGGATGCCGG
DM521_pstBF	Verify point mutation	GATCGCCCTGCTGAATGTCG
DM521_pstBR	Verify point mutation	GAAGTCGCCGTCGGGAAC
DM625_pstSF	Verify point mutation	TACGTTGCCGGGGAGTCTG
DM625_pstSR	Verify point mutation	GTCACTGTTATCCCGTCGGG
DM664_pstCF	Verify point mutation	GCCCAACGCCTTCAAAGAGC
DM664_pstCR	Verify point mutation	GTGTGGTCGGTCAGCCTG

[a]Restriction enzyme sites used for cloning are underlined. Start and stop codons in primers used for construction of in-frame deletions are indicated in bold.

Table S3. Oligonucleotides used for qRT-PCR.

Gene	F primer sequence 5'-3'	R primer sequence 5'-3'
Msm_sigA	CCAAGGGCTACAAGTTCTCG	CCATGTGCACCGGGATAC
Msm_pstS	GGCGTCGACAAGCTGGTACT	GGTGATCTGGCCTTGGAAGA
Msm_regX3	TCCCGTGCGCATGGA	GGCAACGTGATCGGTTCAC
Msm_phoA	CGCAGAAGGCCATCGATCT	ATCGACGCGCCTTCCA
Msm_1605	GCAGACGGCGTTCAAACT	ACGACCATACGCAGCTCACT
Msm_5776	AGAGGTCAACGGCTACTTCG	CGTCGTCTTCCTCCTGGAT
